# Association between Intraoperative Blood Pressure and Postoperative Delirium in Elderly Hip Fracture Patients

**DOI:** 10.1371/journal.pone.0123892

**Published:** 2015-04-10

**Authors:** Nae-Yuh Wang, Ai Hirao, Frederick Sieber

**Affiliations:** 1 Department of Medicine, Biostatistics, and Epidemiology, Johns Hopkins Medical Institutions, Baltimore, Maryland, United States of America; 2 Department of Anesthesiology and Critical Care Medicine, Johns Hopkins Bayview Medical Center, Baltimore, Maryland, United States of America; Massachusetts General Hospital, UNITED STATES

## Abstract

**Background:**

One possible area of intervention to prevent postoperative delirium (PD) is intraoperative blood pressure management. However, the relationship between intraoperative blood pressure and PD is unclear. A secondary analysis of a RCT study examining the PD risk over the range of absolute intraoperative mean arterial blood pressure (MAP) readings and the corresponding relative changes from preoperative baseline level was performed to determine the role of MAP on PD.

**Methods:**

Nonparametric locally weighted quadratic polynomial smoothing (LOESS) regression explored the pattern of PD risk at postoperative day 2 as a function of mean surgery MAP (msMAP) and percent change of msMAP from baseline in 103 elderly hip fracture patients. Segment-linear logistic regression models were then constructed to determine the odds ratios (OR) of PD over the observed range of these msMAP measures, adjusting for potential confounds.

**Results:**

Twenty-three patients (22%) developed PD on day 2. LOESS regression revealed a j-shaped association between absolute levels of msMAP and PD risk. When msMAP was ≥80 mmHg, higher msMAP imparted greater PD risk (OR = 2.28 per 10 mmHg msMAP increase; 95% CI: 1.11–4.70), while higher msMAP was associated with lower PD risk (OR = 0.19 per 10 mmHg increase; CI: 0.05–0.76) if msMAP was <80 mmHg. There was no statistically significant relationship between PD risk and average percent change from baseline in these msMAP measures.

**Conclusion:**

In elderly hip fracture patients, both very high and very low levels of msMAP were associated with significantly increased risk of PD.

## Introduction

Postoperative delirium (PD) is one of the most common complications following surgery in the geriatric population with a reported incidence ranging from 10–70% depending on the type of surgery[1].Delirium is expensive, accounting for an estimated 142–153 billion healthcare dollars in the United States annually[2]. Adverse outcomes associated with PD include poor functional status, decline in cognitive status, increased length of stay and associated complications. Multiple studies have demonstrated that patients with PD are less likely to return to their preinjury level of function, are more frequently placed in nursing homes, and display an increase in associated mortality[3]. Given these facts, it is important to find better means of preventing delirium after surgery.

One possible area of intervention to prevent PD is intraoperative blood pressure management. However, the relationship between intraoperative blood pressure and PD with non-cardiac surgery is unclear. Moller et al. [4] studied 1218 patients older than 60 years of age and found no association between one or more episodes of intraoperative mean arterial blood pressure (MAP) decrease to less than 60% of baseline for greater than 30 min and postoperative cognitive dysfunction (POCD). In contrast, delirium following colorectal surgery has been associated with intraoperative MAP decreases under 60 mm Hg[5]. In 235 total hip arthroplasty patients older than 50 years of age under epidural anesthesia randomized to 45–55 mm Hg MAP vs. 55–70 mm Hg MAP, Williams-Russo et al. [6] found no difference in cognitive function between these two blood pressure groups. Summing the above results for non-cardiac surgery, the evidence from the case series seems inconclusive with one randomized controlled trial [6] suggesting that moderate levels of hypotension may be well tolerated. All of the above mentioned studies used MAP to define blood pressure levels.

In 114 hip fracture patients older than 65 years of age under spinal anesthesia, our group randomized patients to light vs. deep sedation to determine the effect of depth of sedation on postoperative delirium (PD)[7]. However, an in-depth analysis of the effect of intraoperative blood pressure changes on cognition was not performed at that time. In this report, we conducted a secondary data analysis of our trial data to examine the PD risk over the entire range of absolute intraoperative MAP readings as well as the relative MAP changes from preoperative baseline during surgery to inform the role of intraoperative MAP on PD risk in elderly hip fracture patients.

## Methods

### Study Procedures

The research protocol was approved by the Johns Hopkins IRB (NA_00078623). All participants provided their written informed consent to participate in a randomized trial that examined the effects of light vs. deep sedation with propofol in elderly patients during hip fracture repair under spinal anesthesia (trial registered at ClinicalTrials.gov under registration number: NCT00590707). This study is a secondary analysis of data from the completed randomized controlled trial. The primary outcome of the trial was in hospital PD on postop day 2 and the trial protocol was previously described[7].

### Study Population

Patients who were 65 or more years of age undergoing hip fracture repair with spinal anesthesia and propofol sedation.

### Study Protocol

Preoperative screening was performed with the Mini-Mental State Examination (MMSE)[8] and the Confusion Assessment Method (CAM)[9]. Those with severe cognitive impairment (MMSE < 15) and preoperative delirium were excluded. Patients were randomized to receive deep or light sedation. Spinal anesthesia was administered in all patients. During surgery, sedation was provided by a propofol infusion targeted to a bispectral index (BIS) number of approximately 50 in the deep sedation group and a BIS number of approximately 80 in the light sedation group. Postoperatively, analgesic therapy was standardized. Throughout hospitalization, data were obtained on pain (measured on a 0–10 discrete scale), opioid administration, other medications, transfusion requirements, and complications. On the second postoperative day, delirium was assessed by the CAM and MMSE.

### Study data

The outcome of this analysis is PD on day 2 in hospital. The time period for intraoperative blood pressure analysis was the time span of the surgery, which was defined as from incision to end of surgery. Patients with failed spinal anesthesia who had to undergo general anesthesia, and those without complete vital signs data were excluded. MAP was recorded every 5 minutes in the operating room using oscillotonometry (Dinamap). The BP readings between incision and end of surgery were used to calculate the mean surgery MAP (msMAP) values. The average values for all vital signs recorded during the index hospitalization from the emergency room until time of surgery were used as estimate of preoperative blood pressure and was referred to as baseline MAP in this report. Preoperative cognitive impairment was determined by clinical assessment of the attending geriatrician or MMSE score of less than 24. A detailed comorbidity history was collected to construct the Charlson comorbidity index score. Other standard preoperative and intraoperative measures such as time from admission to surgery and units of blood transfused were also recorded.

### Statistics

For each study patient, the average MAP during surgery (msMAP) was calculated as the area under the MAP curve formed by the 5-minute epoch recordings during surgery, divided by the length of surgery, to provide absolute level of mean MAP assessment during surgery. The relative changes of MAP levels from baseline were derived by calculating the ratio of change in msMAP from baseline (msMAP—baseline MAP) to the level of baseline MAP. The ratios were then converted to percentage to form assessments of % change in surgery MAP from baseline. Nonparametric locally weighted quadratic polynomial smoothing (LOESS) plot was utilized to explore the pattern of PD risk as a function of msMAP and percent change of msMAP from baseline. The LOESS results were then used to guide the selection of proper segment-linear logistic regression models that reflect the inflection points on PD risk identified in the LOESS regression, if any, while providing easy to interpret constant odds ratio within the linear (in log-odds) segment on either side of the inflation points, to approximate nonlinear association between PD risk and the predictors. These segment-linear logistic regression models were used for estimating the odds ratios (OR) of PD associated with absolute and relative level of MAP assessments during surgery and their corresponding 95% confidence interval (CI), while adjusting for patient age, preoperative cognitive impairment, depth of sedation, baseline MAP, Charlson comorbidity index score, duration of surgery, and units of RBC transfusion. A p≤0.05 was considered statistically significant.

## Results

The analysis included 103 patients from our previous randomized controlled trial. Ten patients were eliminated from the original trial because of inability to perform spinal anesthesia. One additional patient from the original data set was excluded because of incomplete vital signs data.

The mean (± SD) age was 82±7 years, with mean Charlson co-morbidity index score of 5±3. Seventy-one percent of the patients were female and the overall prevalence of preoperative cognitive impairment was 25%. Twenty-three patients (22%) developed PD on day 2. Population demographics are shown in Table 1. Preoperative MMSE was higher in patients without PD. PD patients received more blood transfusions. Baseline blood pressure measurements were not different between patients with and without PD on day 2.

**Table 1:** PATIENT CHARACTERISTICS, presented as N (%) or MEAN ± SD.

Variable	Total	Postoperative Delirium		P
		Yes (N = 23)	No (N = 80)	
Female	73 (71%)	15 (65%)	58 (73%)	0.60
Age (years)	82 ± 7	84±8	81±7	0.10
BMI (kg/m^2^)	23.9 ± 4.6	23.8±4.3	23.9±4.8	0.92
MMSE	25 ± 5	22 ± 5	26 ± 5	0.01*
Preoperative dementia diagnosis	26 (25%)	11 (48%)	15 (19%)	0.01**
Charlson co-morbidity index	5 ± 3	6±3	5±3	0.09
Number of prescribedMedications preoperatively	4 ± 3	4 ± 3	4 ± 2	0.40
Preoperative hypertension diagnosis	88 (85%)	22 (96%)	66 (83%)	0.18
Baseline SBP (mm Hg)	134 ± 17	136±18	133±17	0.59
Baseline DBP (mm Hg)	70 ± 11	74±14	69±10	0.12
Baseline MAP (mm Hg)	94 ± 15	98±17	93±14	0.20
Baseline HR	78 ± 21	81 ± 12	77 ± 23	0.20
Days to surgery	2 ± 2	2 ± 2	2 ± 2	0.88
Average BIS value during surgery	68 ± 22	59 ± 22	71 ± 21	0.02*
Length of surgery (minutes)	85 ± 37	74 ± 33	88 ± 38	0.08
RBC transfusion (units)	1.0 ± 1.1	1.4 ± 1.2	0.8 ± 1.0	0.05*
Length of stay (days)	7 ± 4	8 ± 6	6 ± 3	0.14

*P<0.05 by t-test;

**P<0.05 by chi-square

**Fig 1:**
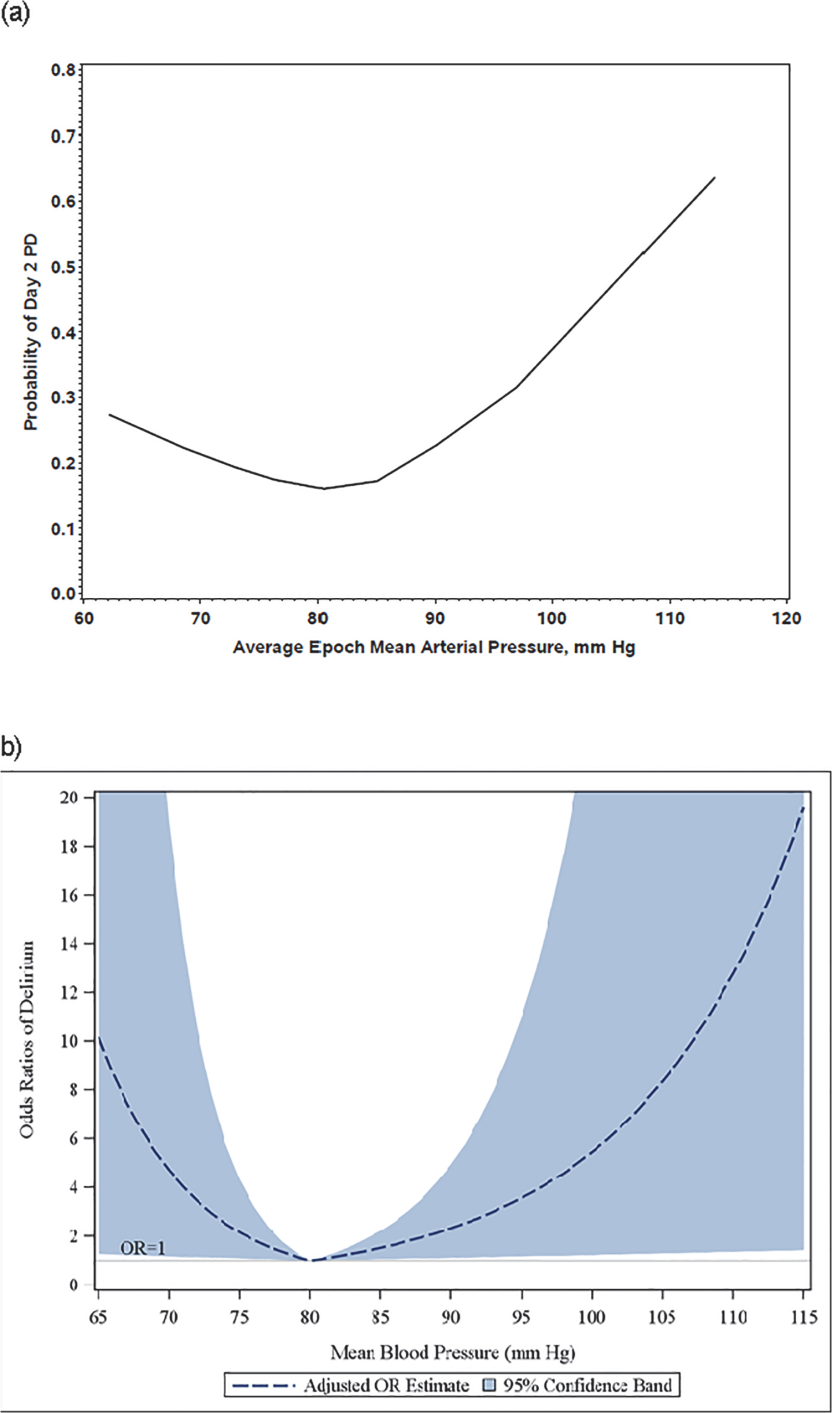
Delirium risk on postop day2 and msMAP. a: Nonparametric LOESS plot of delirium risk on postop day 2 and msMAP. The x-axis denotes the msMAP. The y-axis denotes the risk of delirium on postoperative day 2. b: Adjusted odds ratios of delirium risk on postop day 2 on msMAP after adjustment for age, preoperative cognitive impairment, corresponding BP value at baseline, trial intervention received, Charlson co-morbidity index score, duration of surgery, and unit of RBC transfusion. The x-axis denotes the msMAP. The y-axis denotes the model-based odds ratio of delirium on postoperative day 2.

Nonparametric LOESS plot revealed a J-shaped association between msMAP and PD risk on postop day 2 (Fig 1A). The LOESS results in logit scale (i.e.-log odds) clearly indicated a linear spline with a knot at 80 mmHg as a reasonable logistic regression model for the msMAP-PD association, which describes a change of direction in risk associated with msMAP depending on the level of msMAP. Specifically, the data suggest a linear increase of log odds of PD as msMAP increased if msMAP was in the range of ≥80 mm Hg, while log odds of PD decreased linearly as msMAP increased if msMAP was in the range of <80 mm. Therefore, a segment-linear logistic regression analysis with a linear spline at 80 mm Hg was undertaken, corrected for age, preoperative cognitive impairment, corresponding BP value at baseline, trial intervention received, Charlson co-morbidity index score, duration of surgery, and RBC transfusion (Table 2 and Fig 1B). In this linear spline logistic regression model, every 10 mm Hg increase in msMAP was shown to be associated with a 2.3-fold increase in adjusted odds of delirium on postop day 2 if msMAP was ≥80 mm Hg (p = 0.025) and a 81% reduction in adjusted odds of PD (p = 0.019) for every 10 mm Hg increase in msMAP if msMAP was <80 mm Hg (Table 2). Baseline MAP was not associated with PD after accounting for the corresponding msMAP levels during surgery. There was no association with a % drop in msMAP and PD (see supplementary materials).

**Table 2:** Logistic regression for risk of delirium on postoperative day 2 by increase in msMAP adjusted for age, preoperative cognitive impairment, MAP value at baseline, trial intervention (depth of sedation) received, Charlson comorbidity index score, duration of surgery, and units of RBC transfusion.

	OR(95%CI)	P
Per 10 mmHg increase of msMAP if msMAP was < 80 mmHg	0.21 (0.05–0.86)	0.03
Per 10 mmHg increase of msMAP if msMAP was 80 mmHg or higher	2.34 (1.11–4.94)	0.03
Age	1.09 (0.996–1.19)	0.06
Preoperative cognitive impairment	2.63 (0.67–10.27)	0.16
MAP value at baseline	1.44 (0.92–2.25)	0.11
Trial intervention	0.22 (0.06–0.74)	0.01
Charlson comorbidity index	0.96 (0.78–1.18)	0.69
Surgery duration	0.99 (0.97–1.01)	0.53
RBC transfusion	1.51 (0.92–2.47)	0.11

The breakpoints (knots for linear spline) for the logistic regress models were determined using Fig 1A.

## Discussion

This study shows that for elderly patients undergoing hip fracture repair surgery, very high or very low level of msMAP were both associated with significantly increased risk of postoperative delirium in hospital. For patients with msMAP ≥80 mm Hg, our data suggest a continuing increase in odds of in hospital PD associated with increasing msMAP during surgery and that every 10 mm Hg greater in msMAP was estimated to impart more than 2 fold increase in odds of PD, while for those with msMAP <80 mm Hg lower msMAP seemed to be associated with significantly greater risk of PD. Our results suggest that management of intraoperative hypertension may be as important as managing intraoperative hypotension in optimizing cognitive outcomes in the elderly.

Several studies have reported relationships between perioperative hypotension and neurologic outcomes. In non-cardiac surgical patients, a nested case control study examining the relationship between hypotension and perioperative stroke reported that each minute of hypotension, defined as a 30% change in MAP from baseline, accounts for a 1.3% increased risk of stroke [10]. In the POISE trial clinically significant hypotension defined as SBP below 100 mm Hg for an unspecified period of time was an independent predictor of stroke (OR = 2.14, CI:1.15–3.96) [11]. Given the above, it appears that for stroke in non-cardiac surgical patients, hypotension defined by either absolute or relative decreases, is an important etiologic factor. In terms of PD, previous reports in hip fracture patients show that PD is associated with 30% or greater decrease in blood pressure with spinal anesthesia[12]. When comparing elderly (>75 years) hip fracture patients to younger controls, spinal anesthesia is associated with a greater incidence of hypotension (>20% decrease in SBP) and a statistically significant reduction in middle cerebral artery velocity[13]. Whether or not these reductions in middle cerebral artery velocity are associated with alterations in brain oxygen saturation is unclear. Among elderly patients undergoing surgical fixation of fractured femur decreases in regional brain oxygen saturation do occur, but appear to be patient specific and independent of the anesthetic technique[14]. However, recent studies suggest that decreases in regional brain oxygen saturation may be associated with cognitive dysfunction [15]. Although the current study found no association between moderate average decreases in blood pressure from baseline and PD, both the literature and the current study suggest that larger average decreases from baseline are associated with both increased PD risk as well as stroke.

The current study suggests that intraoperative hypertension is as important as hypotension in determining PD. It is clear that hypertension is associated with poorer cognitive performance[16] and that more severe forms of hypertension, such as hypertensive crisis, may be associated with delirium[17]. There are reported relationships between preoperative hypertension and postoperative decline in cognitive function[18]. Moreover, preoperative hypertension is associated with perioperative hypertensive events[19]. The biological concern in the current study in relation to the BP level associated with low PD risk, is that they appear to be surprisingly low. These msMAP values may be related to the cerebral autoregulatory thresholds observed in patients with underlying vascular disease. A recent study[20] using near infrared spectroscopy technology examining the relationship between upper cerebral autoregulation thresholds and PD reported that the magnitude and duration of MAP above the upper limit of autoregulation in cardiac surgery patients during bypass is an independent risk factor for PD.

The limitations of this study include a relatively small sample size and limited number of observed PD events, which restricted our ability to further account for potential confounding factors through statistical modeling in logistic regression analyses. Therefore, the possibility of residual confounding cannot be ruled out. Post-hoc secondary analyses using trial data are exploratory in nature. No statistical power evaluation was conducted to ensure adequate power for the hypothesis explored in this report, no adjustment was made to account for trial-wise type I error. Inclusion of elderly patients with MMSE 15–20 in our trial may cause bias in the statistical analysis. Although dementia is a key risk factor for postoperative delirium, our RCT demonstrated that response to level of sedation is similar for patient groups with preoperative MMSE 15–20 vs. 21–30[7]. In otherwords, lighter sedation leads to a lower incidence of postoperative delirium. Another reason for including dementia patients is to increase applicability of our findings. Dementia is common in this surgical population, for example patients with MMSE<21 constituted 29% of our study population. Only one type of surgical population was studied, therefore the results cannot be generalized to other surgical populations. The time period of blood pressure measurements studied was from incision to end of surgery. Blood pressure alterations occurring from entry into operating room until incision, in the PACU, and on the ward could have contributed to cognitive changes seen in these elderly patients. In addition, the current study did not allow for analysis of the duration of blood pressure increase/decrease required for PD to occur because the duration examined was dependent on length of surgery (average duration approximately 80 minutes). Baseline MAP represented the average values for all vital signs recorded during the index hospitalization from the emergency room until the patient appeared in the preoperative holding area. However, the calculated baseline values may not be representative because of modifying influences such as pain and anxiety. The bias of one-time delirium assessment on the second postoperative day may have underdetected mild, transient delirium that occurred only on first postoperative day. Previous studies in hip fracture patients report that delirium severity peaks on day one postoperatively[21]. Thus, delirium incidence in the current study may have been under reported. Nonetheless, delirium episodes missed would have been mild. Furthermore, Diagnosis of delirium is difficult in patients with MMSE< 15 so such patients were excluded from the trial. And the original RCT did not further ascertain the severity of preoperative cognitive impairment. Finally, our evaluation of only in-hospital outcome measures does not permit insights into any long-term associations of intraoperative blood pressure management and outcomes. Given these limitations, the finding of this study should be considered hypothesis generating rather than confirmatory. We suggest that further hypothesis generated studies should focus on cerebral autoregulatory thresholds.

In summary, our study shows that in elderly patients undergoing spinal anesthesia for hip fracture repair, very high or very low levels of msMAP were both associated with significantly increased incremental risk of PD. For patients with msMAP ≥80 mm Hg, our data suggest that every 10 mm Hg greater in msMAP was associated with 2.3 fold increase in odds of in hospital PD, and a 81% reduction in adjusted odds of PD if msMAP was <80 mm Hg. Our results suggest that management of both intraoperative hypertension and hypotension are important in optimizing cognitive outcomes in the elderly. Further work is needed in the elderly population to better define the relationships between intraoperative blood pressure changes/management and postoperative cognition.

## Supporting Information

S1 Fig Delirium risk on postop day 2 and % reduction from msMAP. Nonparametric LOESS regression of delirium risk on postop day 2 on % reduction from baseline of mean arterial blood pressures during surgery (msMAP). The x-axis denotes the % msMAP reduction from baseline MAP. The y-axis denotes the risk of delirium on postoperative day 2. Nonparametric LOESS regression demonstrated that there was no association for % change in msMAP from baseline with risk of PD on postop day 2.
(TIF)

S1 Table Logistic regression for risk of delirium on postoperative day 2 by % reduction of intraoperative MAP (msMAP) values from baseline MAP, adjusted for age, preoperative cognitive impairment, MAP value at baseline, and trial intervention received. (DOCX)
