# Supplementary material for: Association between Intraoperative Blood Pressure and Postoperative Delirium in Elderly Hip Fracture Patients
Source: PLoS One. 2015 Apr 10;10(4):e0123892. doi: 10.1371/journal.pone.0123892 (PMC4393126; doi:10.1371/journal.pone.0123892)
Supplement: S1 Table — (DOCX) [file pone.0123892.s002.docx]

S1 Table: Logistic regression for risk of delirium on postoperative day 2 by % reduction of intraoperative MAP (msMAP) values from baseline MAP, adjusted for age, preoperative cognitive impairment, MAP value at baseline, and trial intervention received. There was no association for % change in msMAP from baseline with risk of PD on postop day 2.

|  | OR(95%CI) | P |
| --- | --- | --- |
| Overall-Per 10% drop of msMAP from baseline | 0.93 (0.61-1.42) | 0.75 |
| Per 10% drop of msMAP from baseline if msMAP was higher than baseline | 0.99 (0.45-2.17) | 0.98 |
| Per 10% drop of msMAP from baseline if msMAP was lower than baseline | 0.90 (0.48-1.67) | 0.73 |
